# Tools for communicating risk for Parkinson’s disease

**DOI:** 10.1038/s41531-022-00432-6

**Published:** 2022-11-29

**Authors:** Lola Cook, Jeanine Schulze, Wendy R. Uhlmann, Jennifer Verbrugge, Karen Marder, Annie J. Lee, Yuanjia Wang, Roy N. Alcalay, Martha Nance, James C. Beck

**Affiliations:** 1 Department of Medical and Molecular Genetics, Indiana University School of Medicine, Indianapolis, IN USA; 2 Department of Internal Medicine (Division of Genetic Medicine) and Department of Human Genetics, University of Michigan, Ann Arbor, MI USA; 3 Department of Neurology, College of Physicians and Surgeons, Columbia University, New York, NY USA; 4 Department of Biostatistics, Mailman School of Public Health, Columbia University, New York, NY USA; 5 Department of Neurology, Columbia University Irving Medical Center, New York, NY USA; 6 Neurological Institute, Tel Aviv Sourasky Medical Center, Tel Aviv, Israel; 7 Department of Neurology, Park Nicollet Struthers Parkinson’s Center, Minneapolis, MN USA; 8 Parkinson’s Foundation, New York, NY USA

**Keywords:** Parkinson's disease, Neurology

## Abstract

We have greater knowledge about the genetic contributions to Parkinson’s disease (PD) with major gene discoveries occurring in the last few decades and the identification of risk alleles revealed by genome-wide association studies (GWAS). This has led to increased genetic testing fueled by both patient and consumer interest and emerging clinical trials targeting genetic forms of the disease. Attention has turned to prodromal forms of neurodegenerative diseases, including PD, resulting in assessments of individuals at risk, with genetic testing often included in the evaluation. These trends suggest that neurologists, clinical geneticists, genetic counselors, and other clinicians across primary care and various specialties should be prepared to answer questions about PD genetic risks and test results. The aim of this article is to provide genetic information for professionals to use in their communication to patients and families who have experienced PD. This includes up-to-date information on PD genes, variants, inheritance patterns, and chances of disease to be used for risk counseling, as well as insurance considerations and ethical issues.

## Introduction

Over the past 30 years, variants in the genes *SNCA*, *PRKN, LRRK2*, *GBA1*, and others, have been recognized as important to the etiology of Parkinson disease (PD)^1^. In approximately 10% or more of patients with PD in North America, testing will uncover a major pathogenic variant (manuscript in preparation; LC et al). When inherited, the disease can be autosomal dominant, often with age-related reduced penetrance and, predominantly, later onset, or it can be autosomal recessive, caused by gene variants that are mostly fully penetrant leading to early or young onset^1^. In addition to variants in the major genes listed above, at least 90 PD risk loci have been discovered by genome-wide association studies (GWAS) that contribute to heritability^2^.

In the United States (U.S.), genetic testing for PD is widely available through commercial companies, including direct-to-consumer (DTC) testing, and may be offered at no cost through research studies and biopharma-sponsored testing programs^3^. Research studies like PD GENEration, Parkinson’s Progression Markers Initiative, and others are actively enrolling individuals with PD, with the goal to expand genetic testing, making it more accessible, including outside the U.S^4^. Increasingly neurologists and other health care providers (HCPs) may face questions about PD genetics, genetic test results, and risks. Independently, patients with a family history of PD or individuals, just curious about their risks for adult-onset conditions, may also seek this information.

Genetic communication about PD may be perceived as challenging due to its complex inheritance, yet HCPs can gain confidence in this area by learning basic PD genetics concepts and how to access resources to aid them in risk assessment and counseling, including resources for finding genetic professionals. We provide recommendations for PD genetic risk communication based on our experience of providing genetic counseling to over 10,000 individuals, both affected and unaffected, as part of several large national PD studies (NCT04477785, NCT04994015). Highlighted as well, is the importance of being aware of genetic advances, working collaboratively with HCPs with genetics expertise, and when relevant, referring directly to genetic counselors who are familiar with PD. This is due to the complexities of risk assessment, nuances of risk communication, genetic testing considerations, and ethical issues related to this disorder as outlined in this article. Although this article is targeted primarily to neurologists, especially those working with patients with movement disorders, some of the high-level points and takeaways may be of use to general practitioners. Advanced topics, illustrative cases, and risk calculations may be especially beneficial to PD specialists working in the field.

## PD genetic risk communication put into action

### Setting the stage

Genetic risk communication for PD, as part of formal genetic counseling, can occur in different settings, for various indications—clinical or research, and for predictive, diagnostic, or prenatal reasons. Regardless of the setting, genetic counselors are trained to adhere to the following best practices for risk communication^5,6^.

- Use of plain language
- Provide only important key elements (less is more)
- Keep the discussion interactive
- Avoid framing biases (giving both the positive and negative views)
- Avoid qualifiers such a low, slight, or high risk
- Use absolute rather than relative risks
- Draw attention to the fact that a risk may change over time
- Compare risks to disease prevalence or other baseline comparators
- Consider the use of visual aids especially for those with low numerical literacy

Genetic counselors may see individuals with PD concerns in genetics, prenatal, or neurology clinics, and through PD research initiatives. Due to their expertise, they may also see asymptomatic individuals seeking predictive genetic testing. In contrast, physicians will see mostly symptomatic patients with manifest disease, in a neurology setting, where testing fits into the diagnostic category. Increasingly, they may see individuals with prodromal disease who do not yet meet clinical criteria for a diagnosis. This may be especially true as biomarkers are developed and enter the clinical arena. A distinct and separate group—at-risk, asymptomatic individuals—may be interested in baseline evaluations or simply consultations and may be armed with genetic test results they have procured through DTC testing or through relatives in their family who did genetic testing.

For individuals with PD, the genetics discussion in the neurology setting will center on implications to their care, but may quickly turn to their family members, as has been our experience, as well as that of others^7^. Although risk discussions in neurology are common, genetic risk discussions uniquely shift the unit of care from the patient to the family, often requiring a different approach. This may be especially true when encountering individuals from other cultures where there is a strong emphasis on the family unit^8^. One of the more common questions we are asked by research participants receiving PD genetic testing is what the risk of their children (or other relatives) is for developing the disorder. In the following sections we provide guidance on addressing this and similar questions including those related to causation.

### Risk assessment comes first

Before a provider can provide accurate genetic risk communication, a genetic risk assessment needs to happen. Reviewing a patient’s medical and family histories is the first assessment step before embarking on genetic and risk discussions. A family history that is targeted for neurological conditions that includes two to three generations can be an important risk assessment tool. Ancestry can be another relevant inquiry in PD, since PD variants can vary across populations affecting risk^1,4^. For instance, obtaining family history intake that includes Lewy body dementia may indicate the potential for *GBA1* variants, especially in a family that is Ashkenazi Jewish^1,4^. In addition, it can be helpful to inquire if a patient or someone in the family has had genetic testing and if results are known. It is not uncommon for *GBA1* testing to have been done in a prenatal setting in other relatives as part of carrier testing for Gaucher disease, and positive Gaucher results may not be understood to be connected to the risk of developing PD^9^. To aid in family history intake, short, targeted family history intake questionnaire forms have been developed by researchers for various PD projects and can be HCP- or patient-facing^10^.

A key concept to consider while performing risk assessment is whether the PD is of early or late onset. Given that there are different genetic etiologies and inheritance patterns for early (<50 years) and late-onset PD (≥50 years), age of onset (ideally confirmed diagnosis) should be ascertained. When disease onset is of young onset (30s or younger), this can be due to major genetic factors that are fully penetrant by early or mid-adulthood and, typically, inheritance is autosomal recessive. This contrasts with late-onset disease, which most often is due to a complicated interplay between multiple genes, some rare but mostly common and of variable effect size, as well as environmental factors; thus, it follows a multifactorial pattern of inheritance with higher cumulative risk as age progresses. Those who fall between age ranges can represent either category but often have an earlier manifestation of a late-onset form of PD, sometimes observed with *GBA1* or *LRRK2* forms of PD^7,11^.

## Targeted genetics education without testing

Following risk assessment, targeted education about PD genetics can be provided based on patient background and needs. The following can be briefly explained without knowledge of patient genetic status:

- Basic genetics concepts
- Inheritance patterns
- Options for genetic testing
- Potential value of testing

**Fig. 1:**
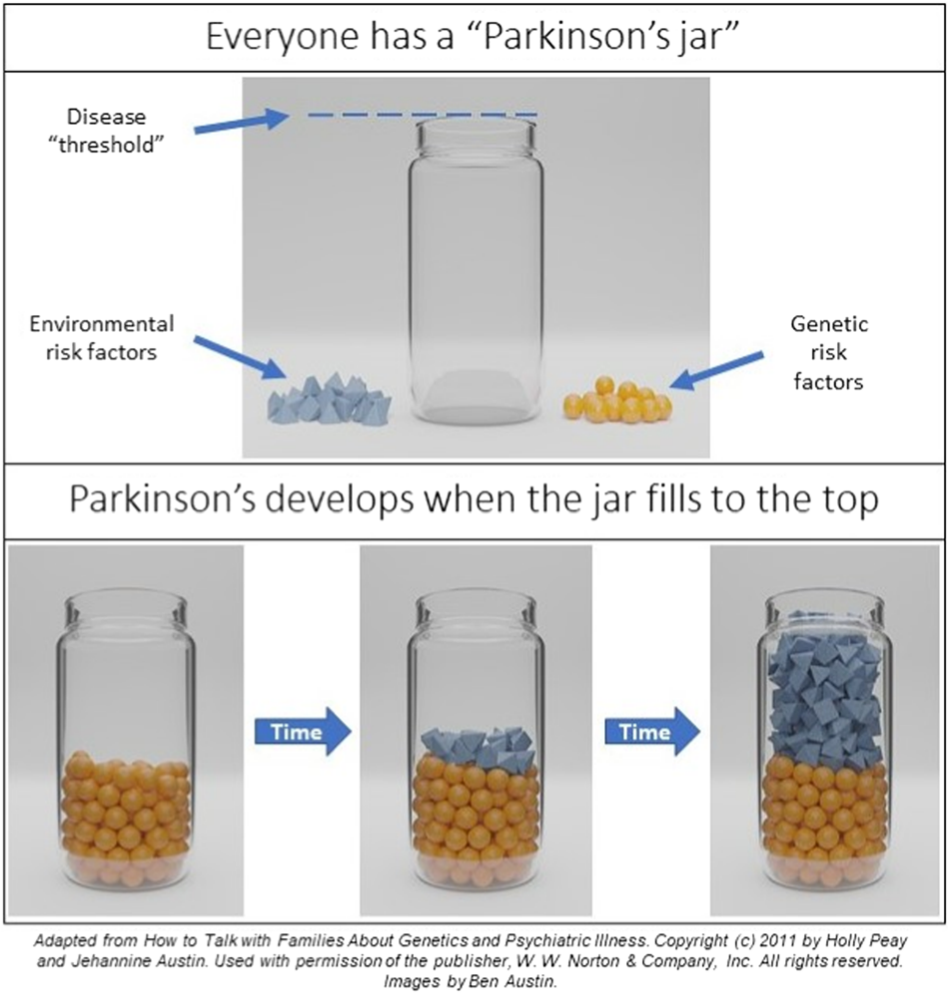
The jar and shapes can help demonstrate the multifactorial model of inheritance to patients. Sample statement: Environmental factors (blue triangles) increase over time and can reach a disease threshold (top of jar). Genetic vulnerability (yellow beads), with which we are born, contributes to the jar, but is mostly static. Lifestyle habits such as moderate aerobic exercise may add “protective rims” to make the jar taller, raising the disease threshold.

Specific talking points can be used, depending on the situation (Table 1), as well as visual aids explaining multifactorial inheritance (Fig. 1) and other forms of inheritance, when relevant. Estimates for relative risk for PD based on degree of relationship to the affected (including up to third degree) are available, ascertained from North American data^12^. In general, the lifetime risk for PD to a first-degree relative of someone with late-onset PD is doubled (Table 2). Lastly, interest, understanding, and perceptions of this information by the patient can be explored.

**Table 1:** Talking points to share with patients with late-onset Parkinson’s disease (PD).

• Most PD is caused by multiple factors, both genetic and environmental, many of which are not yet known.
• Advancing age increases the chance of developing disease.
• Genes that you inherit, from your parents, usually play a role in developing PD.
• Some families have major gene variants, dominantly inherited, that significantly increase the risk for developing PD.
• Current genetic testing can look for some of these major gene variants to aid in risk assessment, risk counseling, and providing a reason for development of the disease.
• However, not all PD genes or changes in known genes can be identified with today’s technology. Therefore, a negative/normal genetic test result does not rule out all genetic factors including the possibility of recurrence in relatives.
• Whether a major gene variant is present or not, environment, age, and other factors can also influence chances of developing disease.

**Table 2:** Quick view for risk counseling.

Counseling scenario	Generalized genetic risk statements
First-degree relative affected	Compared to a baseline lifetime risk of 3% for the general population, this family history increases chances by about twofold or 6% (GeneReviews® Parkinson Disease Overview)
*LRRK2* G2019S test result in at-risk patient of European or Ashkenazi Jewish ancestry	Compared to the baseline lifetime risk … this genetic test result increases chances to about 26%^a^
*GBA1* positive test result in an at-risk patient of any ancestry	Compared to the baseline lifetime risk of … risk is in the range of 10–30%^a^
*PRKN* positive test result (homozygous) in an affected patient	Assuming the variants are on different chromosomes, the theoretical risk to each offspring for early-onset PD depends on the carrier status of the partner; risk for children to be at least a carrier is 100%, parents will be obligate carriers

^a^Risk estimates are taken from data compiled at https://pdnexus.org/docs/professionals/practice-tools/PD-Gene-Penetrance-Table.pdf.

## Genetic testing considerations

PD genetic testing, if pursued or already performed, can inform and fine-tune the risk assessment and education discussed above. Testing, whether narrow or broad, will vary in the usefulness of information provided. The most encountered test will be the gene panel that may include a handful to many genes and variants, more or less established as linked to PD^3^. Many panels, though not all, will include variants associated with both early and late-onset forms of PD. Ideally, for PD, the testing will include deletion and duplication analysis in addition to the latest sequencing technology. Importantly, full sequencing of the *GBA1* gene should be performed, especially in those who are of Ashkenazi Jewish ancestry^3,4^. The most popular DTC tests available in the U.S. for PD tend to be targeted to two major variants, one in *GBA1* the other in *LRRK2* (23andme.com). Research testing options can greatly differ, depending on the goals of the study^4^. Genetic testing programs will vary in the release of variants of uncertain significance (VUS), which can complicate risk counseling, causing confusion to both patient and provider. Comprehensive and newer genetic tests, including polygenic risk scores for PD that combine minor gene variant effects, will likely make their way increasingly into the clinical arena, adding to testing options and information available for counseling^13^. In addition to the scope and techniques of genetic testing, the ethnicity or ancestry of the individual being tested will determine how informative results are^3,4^.

A best practice for genetic testing that directly impacts risk counseling includes first testing the person with the disorder in the family. This allows for clarity in the meaning of results for the patient and other unaffected relatives who may desire testing. When testing is needed from an affected person in the family not especially interested, pressure on decision-making and ethical dilemmas can occur, which will be discussed later in another section. Fully informed consent for genetic testing involves pre-test education and discussion of risks, benefits, and limitations. It is a process and not just a signature on a consent form. There can be challenges and specific considerations for obtaining informed consent when there is cognitive impairment—not uncommon in PD, health literacy limitations, spoken-language differences, psychological stress, and other factors^5^. A full discussion of considerations for genetic testing for PD as relates to risk assessment and communication are outside the scope of this paper, but multiple publications are available to which the reader can refer^4,7,14^.

## Education and risk communication following genetic testing

With genetic data provided from testing, risk estimates considered prior to testing can be revised and recalculated. With the addition of genetic testing, risk counseling becomes more precise and nuanced (Table 2). A corollary is that genetic test results can be personalized and refined with gathered histories on the patient. When estimating genetic risks using test results, it can be helpful to place them within the context of an individual’s age (risk increases over time for PD), ancestry (e.g., European vs Asian, Ashkenazi Jewish), and family history (both maternal and paternal)^7^.

### Residual PD risk due to negative genetic test results

In both sporadic and familial forms of PD, in most populations, genetic results for major variants are usually negative^11^. Risk communication is still important for negative results since misperceptions may occur^15^. One commonly held misperception of patients is that a negative result on genetic testing means that there is not a genetic contribution or, even, that they do not have PD. Patients may infer that their children will not be at risk to develop disease. This will require clarification, with the reminder that typically PD is caused by multiple factors, including minor or unknown genetic ones not currently tested. An estimation of residual risk to relatives and asymptomatic patients following negative genetic testing can be provided based on available clinical and family histories and the scope of testing performed (refer to Table 2/Case 1 in Supplement). A reminder to the patient regarding how a PD diagnosis is made can also be helpful.

Negative genetic test results when the testing is narrow in scope or lacking can falsely reassure families. Another important caveat to communicate is that genetic data for PD have been collected mostly on European populations^16^. The result is that PD genetic test results and lab interpretations that appear “normal” may not be as informative for those from non-European populations.

### Monogenic gene variants and PD risk

PD genetic test results, when positive, will commonly involve the major genes *LRRK2*, *GBA1*, and *PRKN*^11^. For these three genes and the other established PD genes, principles of monogenic inheritance will apply (Table 3) with some exceptions such as reduced penetrance and variable expressivity. Being familiar with these concepts (Table 4) and the various types of inheritance and associated risks—autosomal dominant, autosomal recessive, multifactorial and, less so, X-linked—will be important for risk assessment and communication. When a gene variant does not follow a classic Mendelian pattern of inheritance, featuring reduced penetrance, risk estimates for a condition are most often probabilities. Typically, population or empirical data are the beginning point to consider for an a priori genetic risk. For neurodegenerative disorders like PD, these are typically observed data in a population across an interval of time, and often will be expressed as a cumulative risk of disease to a specific age^17^.

**Table 3:** Established monogenic PD genes (ordered alphabetically and by inheritance)^a^.

Gene	Inheritance	Risk considerations
*GBA1*^b^	AD	• More common in those w/ AJ ancestry • Cognitive impairment or dementia may be present • Can have earlier onset • Family history of GD may be observed since *GBA1* is also associated with this AR disorder • Both mild and severe variants described • Almost all pathogenic variants are associated with both PD and GD, although there are exceptions • Reduced penetrance
*LRRK2*	AD	• More common in those w/ AJ or N. African Berber ancestry • Primary variant in Europeans is G2019S • Reduced penetrance
*SNCA*	AD	• First reported in a few Italian and Greek families; rare • Can have early onset • Duplications are common
*VPS35*	AD	• Rare • Can have earlier onset
*DJ1*	AR	• Rare • Early onset typical • Unclear if single variants increase PD risk
*PINK1*	AR	• Rare • Early onset typical • Unclear if single variants increase PD risk
*PRKN*	AR	• Most common AR PD gene • Early onset typical • Unclear if single variants increase PD risk • Deletions and duplications common

*AD* autosomal dominant, *AJ* Ashkenazi Jewish, *AR* autosomal recessive, *GD* Gaucher disease, *PD* Parkinson’s disease.

^a^Adapted from Table 1 in “Parkinson Disease Overview” by Cook Shukla L, Schulze J, Farlow J, et al. 2004 May 25 [Updated 2019 Jul 25]. In: Adam MP, Ardinger HH, Pagon RA, et al., editors. GeneReviews® [Internet]. Seattle (WA): University of Washington, Seattle; 1993–2022. Available from https://www-ncbi-nlm-nih-gov.proxy.ulib.uits.iu.edu/books/NBK1223/.

^b^Disagreement over whether *GBA1* variants are risk factors or are monogenic related to their degree of penetrance.

**Table 4:** Important genetic terms for PD risk communication.

Reduced penetrance
• When the probability of manifesting a disease for a particular genotype is <100% (typically defined as a lifetime risk) • Relevant for autosomal dominant variants in *LRRK2* and *GBA1*
Variable expression
• Describes the severity of expression of a disorder among individuals with the same genotype • Genetic forms of Parkinson’s disease can be variable between and within families.
Residual risk
• The chance of developing a condition following a negative test result. This is due to the limitations of most genetic tests to capture all possible gene variants related to disease.
Allele
• An allele is one of two alternative forms of a given gene • Heterozygous (variant affects only one allele) vs homozygous (identical variants on both alleles) vs compound heterozygous (both alleles have variants that are different)

Another consideration is that variants in the same gene may have a different penetrance^1^. The use of up-to-date, accurate population data for a specific gene variant is vital for calculating risk estimates. When using penetrance data, note that data ascertainment, sample sizes, and population biases may erode quality of the data^17^. Another limitation of the data is that it is not entirely clear how various demographic features modulate PD genetic risks such as ancestral background or gender. Another limitation is that some penetrance data, especially for *GBA1*, are based on case-control studies providing only odds ratios (ORs). If used, ORs and relative risks (RR) should be converted to absolute risk estimates for the patient^18^.

Family histories are invaluable in that they may provide additional clues as to how variants have been expressed in a particular family, add insight into the potential for other shared factors, providing personalized risks. As of yet, risk calculators for neurodegenerative disorders such as PD are not widely available for clinical use to combine all the different factors, genetic and non genetic, that contribute to an individual person’s overall risk for disease^18^. Various algorithms such as PREDICT-PD and prodromal criteria have been developed^19,20^, focusing on early clinical features of PD, which may inform counseling as the tools become more refined and are able to incorporate more genetic data including data from non-European populations.

### Complexities related to types of positive genetic test results

PD genetic test results can be complicated, depending on the type of result. For instance, there has been the observation that carriers of autosomal recessive variants may have a slightly increased risk for later-onset PD; however, research continues in this area and conclusions are mixed^21,22^. In the meantime, it can be conveyed that there is not a definite answer regarding risk to those carrying an autosomal recessive PD gene variant, but, if confirmed, is likely of low magnitude and of later onset.

Generally, when two pathogenic PD variants are identified, this is consistent with autosomal recessive inheritance. However, another complexity, similar to that of other autosomal recessive genetic disorders, is whether the variants are on the same (*cis*) or different (*trans*) chromosomes, since this is not typically revealed by genetic testing. Determining the phase, cis or trans, is important since a cis phase would mean that both variants are on the same chromosome and inherited from one parent, versus trans where each chromosome in the pair would have a variant, with one variant copy inherited from each parent. If phase of the variants cannot be readily determined by clinical information, this would be important to clarify with additional testing of parents/other affected family members since it will impact the recurrence risks provided.

Another complex type of PD result is copy number variants such as rare gene duplications and triplications of the *SNCA* gene and deletions and duplications commonly observed for the *PRKN* gene. Providers may be less familiar with these types of gene variants; however, these unique variants follow the same Mendelian rules for monogenic disease^1^.

Finally, the vast number of pathogenic gene variants (400+), and the potential for recombinant alleles, reported for the *GBA1* gene pose their own challenges to risk communication, requiring special attention. Some of these rarer variants have limited empirical data from which to base risk estimates^7^. For most *GBA1* variants, there is the association with Gaucher disease, an autosomal recessive lysosomal disorder that can manifest in childhood or be present unknowingly in mildly expressing adults and be amenable to treatment^9^. For a few *GBA1* variants, some quite commonly carried, such as the E326K variant, this is not the case: Gaucher disease is not caused by carrying biallelic *GBA1* variants, even though the variant is thought to be associated with PD^23^. This additional aspect of the *GBA1* gene, namely its association with Gaucher disease, impact on reproductive risk, and potential medical action, requires added time to discuss and creates complexity to risk communication^9^.

## Risk communication resources for the clinician

### PD variant penetrance data and interpretation for monogenic PD genes

In Table 2, we provide a quick view of general risk statements for the most encountered situations in various PD settings. In addition, other useful resources for PD risk estimates can be found at GeneReviews, an online database that provides up-to-date summaries for specific PD genes, as well an overview. In addition to these resources, HCPs can now access estimates of PD genetic risk, based on current and reviewed published data, in one location, the Disease Penetrance Table (https://pdnexus.org), developed by PD genetic counselors at Indiana University^14^. In this penetrance table, the data for each variant type are paired with general statements that can be used in PD risk communication. This information can be used for counseling individuals at risk and shared with affected patients who have questions about risks to offspring, siblings, parents, and other close relatives. It can be discussed with the patient the importance of sharing genetic risk information with key relatives. A family sharing handout that is patient–facing is available for downloading from this website.

It was mentioned, earlier, that visual aids can be instructive when communicating genetic risk. When considering the use of visual aids to enhance risk counseling, it is suggested the number and type of visual aids used should be carefully considered. For example, it is observed that icon arrays can be helpful in displaying absolute risks. Various online sites offer free access to templates to create risk communication graphics. A combined table of resources for PD risk assessment and communication, especially as relates to positive gene test results, are provided for the clinician for easy access (Table 5).

**Table 5:** Parkinson’s disease (PD) genetic risk communication resources for the clinician.

Resources	Details
GeneReviews® *National Center for Biotechnology* https://www.ncbi.nlm.nih.gov/books/NBK1116/	Updated and accurate gene summaries/overviews targeted for the clinician
Indiana University PD Nexus website https://pdnexus.org/	Site developed by IU PD genetic counselors; contains professional risk counseling tools, patient handouts, and other counseling resources
*Disease Penetrance Table* Located under “Professionals: Tools for Your Practice/Risk Assessment”	Provides summary statements for gene-specific risk counseling; reviewed by co-authors K.M., A.J.L., and Y.W.
*Family sharing handout* Located under “Professionals: Tools for Your Practice? Risk Assessment”	Guidance for patients regarding sharing test results
*Genetic Information Nondiscrimination Act (GINA) information* Located under “Professionals: Other Resources”	Detailed information for clinicians and their patients about the federal law GINA
Risk calculator https://clincalc.com/Stats/ConvertOR.aspx	Helpful tool to convert odds ratios into relative risks
Risk communication graphics https://cbssm.med.umich.edu/how-we-can-help/tools-and-resources/pictographs-icon-arrays	Free icon array template developed by the University of Michigan Medical School Center for Bioethics and Social Science in Medicine
Referrals for Genetic Counseling	
NSGC (National Society of Genetic Counselors) Find a Genetic Counselor directory https://www.nsgc.org/page/find-a-genetic-counselor	Directory of registered genetic counselors in the U.S. and Canada, allows filtering by location and specialty
American Board of Genetic Counseling (ABGC) Finding a Certified Genetic Counselor directory https://www.abgc.net/about-genetic-counseling/finding-a-certified-genetic-counselor/	Directory of ABGC Diplomates, certified, who choose to have their genetic counseling services listed; allows filtering by location and specialty

Specific cases, presented below, are provided for the clinician that further illustrate risk assessment and risk communication for various scenarios in the genetic counseling session:

Case 1: Negative Targeted Test Result A 68-year-old Chinese man diagnosed with classic PD is coming to clinic for a follow-up visit. His adult son is accompanying him today and has questions about his own risk for PD. The son mentions his dad had DTC genetic testing for PD, for 2 genes, and they recently learned that his father’s results were normal. He provides a copy of his father’s report and states “This means my father’s Parkinson’s is not genetic, right?”

- Note that relatives may accompany patients at visits asking additional questions relating to their PD risks.
- Question posed represents a common misunderstanding that a negative test result eliminates the possibility of a genetic contribution to disease
- Highlights that DTC testing may be narrow in scope and not necessarily be relevant for a particular population
- May explore the need for additional testing to provide a more precise risk estimate with limitations noted
- A brief family history can be obtained asking which relatives and how many are affected with PD or related conditions and empirical data shared (can quote a background lifetime risk estimate of 3% for a person to develop PD if they live to about 80 years that is increased to about 6% based on population data looking at occurrence in first-degree relatives, see Table 2)
- Visual aids (e.g., Fig. 1) to show numerical risks and the influence of other factors in causing PD can be helpful
- If son has questions relating to his risk for PD, he can be referred for genetic counseling (See Table 5)
- Be aware of patient needs and cultural aspects of this case; for example, Asian Americans may possess an attitude of filial piety—reverence given to parents by their children by caring for them and carrying out their wishes^8^.

Case 2: *GBA1* Positive Panel Result A 50-year-old man of Hispanic ethnicity was diagnosed with PD a year ago. He reports a family history of PD; his mother was diagnosed with PD at age 56, had severe progression and “dementia” later in her disease. He recently had testing done as part of a research study and has genetic questions related to his report. His results from panel testing that includes full sequencing of all genes show that he carries one *GBA1* L444P variant. He has 2 children, under 18, both healthy, and asks about testing for them. He also has five siblings, ages 40–60, two of whom are at the visit today.

- Prior to risk counseling verify the completeness of PD testing performed and if the research testing was done by a CLIA-accredited laboratory. (Testing may not include full sequencing of *GBA1* or include other key genes related to PD, which is relevant to risk counseling.)

- When approaching databases and literature, it is helpful to know the various names used for a *GBA1* variant, in this case, a common, historic one.
- It is important to use specific data for a given variant when available — in this case a variant classified as severe; different *GBA1* variants confer divergent risks. Risk of severe progression and cognitive impairment are increased with certain *GBA1* variants, albeit PD severity is highly variable even among family members with the same variant. Sharing that family members will experience PD in their own way can be a relief to a patient who witnessed a severe form of PD in a close relative, such as this patient.
- Locate up-to-date penetrance data and, ideally, data specific for a variant to provide an accurate risk estimate of developing PD to counsel the patient or other relatives attending the session (see Table 5). The general risk range of 10–30% can easily be remembered for counseling for any *GBA1* variant^7^, stating that for a severe variant the risk approaches the upper end of the risk range.
- To calculate a specific variant risk, the provider can use the OR published for the *GBA1* variant (when available), convert it to a relative risk, using a risk calculator (see Table 5), and then derive an absolute risk, which is more understandable to patients.
- Establish expectations for risk discussion that will include multiple concepts, some complex, and family members. Additional visits including outside genetics referrals for unaffected relatives may be necessary. Be aware of patient needs and cultural aspects of this case — family is typically integral to those in the Hispanic community (familism); often they are involved in decisions that relate to the patient including treatment, care, and interest in research^8,24^.
- For *GBA1* variants, it can be helpful to discuss, first, implications for PD, starting with those things relevant to the patient and then moving to a larger discussion surrounding the familial risk. Key concepts are autosomal dominant inheritance, reduced penetrance, variable expression, and multifactorial etiology.
- Beyond discussion of PD risks for this *GBA1* variant, the implications to family planning, if relevant, and risk of Gaucher disease should be discussed, most relevant to those of reproductive age. Explaining what Gaucher disease is, the concept of autosomal recessive inheritance, and carrier status will be important to convey, as well as the concept of prenatal carrier screening. Referral to a prenatal genetic counselor may be warranted for those of reproductive age (See Table 5).
- If there are concerns for Gaucher disease in any relative, a recommendation should be made to alert their primary physician for further evaluation.
- Visual aids for various parts of the discussion could be useful.
- Note genetic testing of minors is generally not recommended for adult-onset conditions^25^.

## The genetics referral

When dealing with complex genetic issues of the affected patient, asymptomatic patients, relatives at risk, prenatal issues, or complicated genetics test results, a referral to a genetics expert is warranted. This is also the case if the risk discussion is beyond the comfort level of the HCP. It is important to have established professional relationships with genetic counselors on site or locally, so that when these indications do arise, a genetics referral can be easily made (Table 5). Genetic counselor specialties that will likely be most useful to PD patients and their families are as follows: neurology, adult genetics, general genetics, and prenatal centers for individuals planning families. Alternatively, providers can make use of remote genetic counseling services that are expanding nationwide and globally, offer services regardless of residence, and potentially affordable.

## Ethical considerations of genetic testing and risk communication

There are several different ethical issues that can arise with neurogenetic conditions involving testing, risk assessment and communication^5,6^. It should be stressed that the core ethical considerations (e.g., autonomy, beneficence) in genetic counseling are generally approached from predominantly Western cultural values and that other cultures may have different views, especially as it relates to the patient^26^. Ethical issues can arise when there are conflicting views between the patient and at-risk relatives (e.g., children, siblings) about genetic testing and with patients and providers when requesting genetic testing that is contrary to guidelines/standards of care (e.g., requests to test minors or a pregnancy when it will not impact reproductive decisions). Clinicians can find guidance on these issues looking to literature developed for predictive genetic testing for Huntington disease^27^, in existence since the 1980s, and adapted for other hereditary neurological conditions. This guidance emphasizes that it should be the patient’s decision to be tested and there should not be coercion. This means that the patient’s decision about testing needs to be respected and the patient should not be pressured to be tested or not tested.

There can be conflict and potentially ethical issues when a patient’s desire to test or not test is contrary to the desires of other at-risk family members. It can be challenging to determine whose rights take precedence when weighing a patient’s right to know or not know^28^. For at-risk family members, their ability to be tested may depend on knowing whether an affected family member has an identifiable pathogenic variant. DNA banking is an option that respects an individual’s right not to know, yet, at the same time, allows an individual’s DNA to be preserved for future testing.

Although it is recommended that neurologists refer for predictive testing, it can be helpful to be aware of published guidelines including those for Huntington disease genetic testing^27^, and statements from professional organizations, including the American Academy of Pediatrics and the American College of Medical Genetics^25^, the American Society of Human Genetics^29^ and the National Society of Genetic Counselors, who have agreed that predictive genetic testing for adult-onset conditions should not be done on minors. With adult-onset neurological conditions, there is no clinical indication to test children since results would not impact their medical care in childhood. Minors are also not tested to preserve their autonomy and right to an open future. Furthermore, testing a pregnancy for an adult-onset condition should not be done if the results will not impact reproductive decisions since continuance of a positive pregnancy would be akin to testing a minor^30^. As expected, there has not been a strong interest in prenatal or preconception testing for PD documented. Thus, providers should avoid making assumptions about a patient’s interest in pursuing prenatal or preconception testing for PD, and this should be explored on a case-by-case basis with appropriate referrals to genetics professionals if considering this testing.

Patients with PD often express concerns about insurance coverage and the potential of genetic discrimination. The Genetic Information Nondiscrimination Act (GINA) is an important U.S. law passed in 2008 that provides protection from health insurance and employment discrimination related to genetic test results and family history. However, there are some significant limitations in protections; genetic test results may affect obtaining life, disability, and long-term care insurance. There are online resources available for talking about the risk of genetic discrimination, key points to convey, and information that that can be downloaded and provided to patients (Table 5). Although these are important considerations for asymptomatic individuals, it can be explained that they are less relevant for those who are already diagnosed with the condition.

While, ultimately, genetic test results are the patient’s private and confidential information, the fact that results could impact the risk provided to other family members and potentially have implications for their care needs to be conveyed. This is especially true for *GBA1* variants and autosomal recessive PD variants, such as *PRKN*, that are potentially medically actionable given the reproductive implications and, in the case of *GBA1*, the association with Gaucher disease. In the event that a patient refuses to disclose to family members their diagnosis of PD and genetic test results (if tested), the healthcare provider does not have a duty to warn at-risk family members but does have a duty to inform the patient about potential risks to their relatives^31^.

Another ethical consideration is whether there is a duty to recontact when new genetic tests become available, impacting risk assessment and counseling. Advances will continue to be made in understanding the genetics of conditions like PD and genetic test results that may be uncertain for now. Especially for patients with VUS or negative genetic test results, it will be important to emphasize the importance of recontacting their genetics clinic and/or healthcare provider to obtain updates or determine whether newer or additional genetic testing should be considered. The American College of Medical Genetics and Genomics issued a statement that the duty to recontact to learn about genetic advances needs to be a shared responsibility and is not solely the responsibility of the healthcare provider^32^. If new genetic information is obtained by the individual or their family, it would be important to emphasize the sharing of such information so that updates of prior risk estimates can be provided.

A last important point, considering the principles of equity and justice, is that, historically, mostly European populations have been included in genetic and genomic databases. Lack of inclusion of other ancestral groups has limited our knowledge about the genetic contribution to PD in those underrepresented. Thus, genetic test results and interpretations may have reduced accuracy and utility across certain populations^33^. This should be acknowledged by clinicians as risk assessment, communication, and counseling on PD genetics are provided and testing becomes more widespread for PD. Hopefully, the work performed by GP2 and others will begin to address these gaps in PD genetic data, allowing for genetic information to be useful and relevant for underserved and underrepresented populations.

In conclusion we have established that genetic risk communication is different than other types of risk communication in the neurology setting, and this applies to PD. Genetic data are not confined to the individual patient but ripple out to others in the family. Questions can arise surrounding the risk of occurrence and recurrence of PD in individuals and families. Emotionally charged questions such as “Do I need to worry about my children getting this?” deserve good risk communication and triage, regardless of whether a person has received genetic testing.

We have illustrated how PD genetic test results can be complex, featuring both autosomal recessive and autosomal dominant gene variants, including many that act within a multifactorial/complex mode of inheritance. Additional complexities are the need to consider copy number variants and recombinant alleles, reduced penetrance, and the association of *GBA1* variants with Gaucher disease. We recommend that HCPs first assess their comfort level with PD genetics and risk communication. In addition, they will need to assess the needs and background of the patient as it relates to risk information. If providing their own risk counseling, providers can adapt best practices as outlined in this article, using resources developed by experts in the field. We recommend adapting risk estimates to medical and family histories, demographics, and other risk assessments, for a personalized touch. It is important for HCPs to acknowledge the gap in genetic data and interpretation for non-European populations. Finally, neurologists will benefit from collaborating with HCPs trained in PD genetics, as well as genetic counselors, as they navigate discussions surrounding PD genetics and risk.

### Reporting summary

Further information on research design is available in the Nature Research Reporting Summary linked to this article.

## Supplementary information

Reporting Summary

## Data Availability

Data sharing is not applicable to this article; no datasets were generated or analyzed during the current study.

## References

[1] Kim CY, Alcalay RN (2017). Genetic forms of Parkinson’s disease. Semin. Neurol..

[2] Nalls, M. A. et al. Identification of novel risk loci, causal insights, and heritable risk for Parkinson’s disease: a meta-analysis of genome-wide association studies. *Lancet Neurol*. **18**, 1091–1102 (2019). 10.1016/S1474-4422(19)30320-5 PMC8422160 31701892

[3] Cook L (2021). The commercial genetic testing landscape for Parkinson’s disease. Parkinsonism Relat. Disord..

[4] Cook L (2021). Genetic testing for Parkinson disease: are we ready?. Neurol. Clin. Pract..

[5] Roberts JS, Patterson AK, Uhlmann WR (2020). Genetic testing for neurodegenerative diseases: ethical and health communication challenges. Neurobiol. Dis..

[6] Lautenbach, D. M., Christensen, K. D., Sparks, J. A. & Green, R. C. Communicating genetic risk information for common disorders in the era of genomic medicine. *Annu. Rev. Genomics Hum. Genet.* **14**, 491–513 (2013). 10.1146/annurev-genom-092010-110722 PMC3862080 24003856

[7] den Heijer JM, van Hilten JJ, Kievit AJA, Bonifati V, Groeneveld GJ (2021). Experience in genetic counseling for GBA1 variants in Parkinson’s disease. Mov. Disord. Clin. Pract..

[8] Schwartz SJ (2010). Communalism, familism, and filial piety: are they birds of a collectivist feather?. Cult. Divers. Ethn. Minor Psychol..

[9] Cook L, Schulze J (2017). Connecting Gaucher and Parkinson disease: considerations for clinical and research genetic counseling settings. J. Genet. Couns..

[10] Verbrugge J (2021). Outcomes of genetic test disclosure and genetic counseling in a large Parkinson’s disease research study. J. Genet. Couns..

[11] Skrahina V (2021). The Rostock International Parkinson’s Disease (ROPAD) study: protocol and initial findings. Mov. Disord..

[12] Savica R, Cannon-Albright LA, Pulst S (2016). Familial aggregation of Parkinson disease in Utah: a population-based analysis using death certificates. Neurol. Genet..

[13] Hill, E. J. et al. Genome sequencing in the Parkinson disease clinic. *Neurol. Genet.* **8**, e200002 (2022). 10.1212/NXG.0000000000200002 PMC9210549 35747619

[14] Cook L, Schulze J, Naito A, Alcalay RN (2021). The role of genetic testing for Parkinson’s disease. Curr. Neurol. Neurosci. Rep..

[15] Hoell C, Aufox S, Nashawaty N, Myers MF, Smith ME (2021). Comprehension and personal value of negative non-diagnostic genetic panel testing. J. Genet. Couns..

[16] Global Parkinson’s Genetics Program. (2021). GP2: The Global Parkinson’s Genetics Program. Mov. Disord..

[17] Lee AJ (2017). Penetrance estimate of LRRK2 p.G2019S mutation in individuals of non-Ashkenazi Jewish ancestry. Mov. Disord..

[18] Roberts JS, Patterson AK, Uhlmann WR (2020). Genetic testing for neurodegenerative diseases: ethical and health communication challenges. Neurobiol. Dis..

[19] Noyce AJ (2017). PREDICT‐PD: An online approach to prospectively identify risk indicators of Parkinson’s disease. Mov. Disord..

[20] Berg D (2015). MDS research criteria for prodromal Parkinson’s disease. Mov. Disord..

[21] Lubbe SJ (2021). Assessing the relationship between monoallelic PRKN mutations and Parkinson’s risk. Hum. Mol. Genet..

[22] Zhu W (2022). Heterozygous PRKN mutations are common but do not increase the risk of Parkinson’s disease. Brain.

[23] Huang, Y., Deng, L., Zhong, Y. & Yi, M. The association between E326K of GBA and the risk of Parkinson’s disease. *Parkinsons Dis*. **2018**, 1048084 (2018). 10.1155/2018/1048084 PMC5901859 29808112

[24] Damron, L. et al. Hispanic perspectives on Parkinson’s disease care and research participation. *J. Alzheimers Dis*. 10.3233/JAD-210231 (2021). 10.3233/JAD-210231 PMC8203231 33843687

[25] Ross LF, Saal HM, David KL, Anderson RR (2013). Technical report: ethical and policy issues in genetic testing and screening of children. Genet. Med..

[26] Jamal L, Schupmann W, Berkman BE (2020). An ethical framework for genetic counseling in the genomic era. J. Genet. Couns..

[27] MacLeod R (2013). Recommendations for the predictive genetic test in Huntington’s disease. Clin. Genet..

[28] Berkman B. E. Refuting the right not to know. https://digitalcommons.law.umaryland.edu/jhclp/vol19/iss1/2 (2017).

[29] Botkin JR (2015). Points to consider: ethical, legal, and psychosocial implications of genetic testing in children and adolescents. Am. J. Hum. Genet..

[30] Hercher L (2016). Prenatal testing for adult-onset conditions: the position of the national society of genetic counselors. J. Genet. Couns..

[31] ASHG statement. Professional disclosure of familial genetic information. The American Society of Human Genetics Social Issues Subcommittee on Familial Disclosure. *Am. J. Hum. Genet.* **62**, 474–483 (1998). PMC1376910 9537923

[32] David KL (2019). Patient re-contact after revision of genomic test results: points to consider—a statement of the American College of Medical Genetics and Genomics (ACMG). Genet. Med..

[33] Marchant G, Barnes M, Evans JP, LeRoy B, Wolf SM (2020). From genetics to genomics: facing the liability implications in clinical care. J. Law Med. Ethics.

